# A Case of Lactation Anaphylaxis

**DOI:** 10.7759/cureus.5497

**Published:** 2019-08-27

**Authors:** Richard Pescatore, Sumaya Mekkaoui, Brian Duffell, Ralph Riviello

**Affiliations:** 1 Emergency Medicine, Crozer-Keystone Health System, Chester, USA; 2 Emergency Medicine, Crozer-Keystone Health System, Upland, USA

**Keywords:** anaphylaxis, lactation, allergy, breastfeeding

## Abstract

We describe the case of a patient who presented multiple times to the emergency department (ED) with recurrent episodes of anaphylaxis in the immediate postpartum period. It was initially thought to be idiopathic in nature on previous visits, but was ultimately diagnosed as lactation anaphylaxis and successfully managed. The diagnosis was suspected when the detailed history revealed recurrent allergic symptoms with each episode of breastfeeding. Following emergency treatment of anaphylaxis, the patient was advised to transition to formula feeding and had no further allergic episodes from the time of discharge to the three-month follow-up period.

## Introduction

We report a rare and unusual case of lactation anaphylaxis, a life-threatening allergic reaction precipitated by breastfeeding in postpartum woman. Lactation anaphylaxis is extremely rare, and reported only sparingly in the literature [1]. Unique features of this case include the patient’s multiple similar presentations without any identifying triggers as well as the severity of her illness. The patient had a resolution of symptoms with cessation of breastfeeding and transition to formula feeds. We discuss the proposed mechanism of lactation anaphylaxis and a detailed description of the patient’s presentation as a reminder for emergency physicians to consider lactation anaphylaxis in the differential diagnosis of postpartum patients presenting with an allergic reaction.

## Case presentation

A 38-year-old G1P1001 female, three weeks postpartum, presented for the third time in two weeks to the emergency department (ED) with complaints of shortness of breath, wheezing, vomiting, and rash. The patient had presented similarly on her previous visits, was diagnosed with anaphylaxis and treated each time with the appropriate and standard of care and medication regimen, including epinephrine, diphenhydramine, famotidine, prednisone, and intravenous fluids. She was observed overnight and discharged with no obvious allergic trigger identified. On her third presentation, during which the diagnosis of lactation anaphylaxis was ultimately made, she was noted to have diffuse urticaria, respiratory distress, and wheezing. Additionally, the patient was hypotensive with an initial blood pressure of 82/62 mmHg, consistent with a diagnosis of anaphylactic shock. She was again treated with the standard cocktail of medications, which led to the resolution of her symptoms; however, upon further questioning, it was revealed that she had been developing a rash with each episode of breastfeeding and that her profound symptoms requiring ED presentation had always developed following breastfeeding.

The patient was observed for six hours with a presumed diagnosis of lactation anaphylaxis. Symptoms did not recur, and the patient was discharged with the recommendation to cease breastfeeding and switch to formula feeds. At three-month follow-up, the patient reported no further episodes of allergic reaction following the transition to formula feeding.

## Discussion

Lactation anaphylaxis is an extremely rare condition thought possibly to be due to the abrupt withdrawal of progesterone and subsequent estrogen predominance in the immediate postpartum period, leading to mast cell instability and degranulation of mast cells [1]. While poorly understood, the roles of oxytocin, prolactin, adrenocorticotropic hormone, corticotropin-releasing hormone, and increased numbers of antepartum mammary and uterine mast cells have also been questioned [2]. The most accepted mechanism is likely to be immunoglobulin E (IgE)-independent with a simultaneous prolactin surge, which is thought to be responsible for mast cell degranulation. The diagnosis of lactation anaphylaxis is primarily clinical, and the utility of skin prick testing with breast milk and measurement of serum IgE and tryptase levels remains controversial [3-4].

Lactation anaphylaxis has been reported in only a handful of case reports. It is typically recognized only upon detailed history taking, and can be easily missed or misdiagnosed as idiopathic anaphylaxis when the breastfeeding trigger is not elucidated. Early recognition and intervention of anaphylaxis is critical and can be lifesaving. The treatment includes standard antihistamine, glucocorticoid, and epinephrine therapy [5]. Identification and cessation of the breastfeeding trigger can be curative, though prophylaxis prior to breastfeeding with oral antihistamines has also been described as a successful management strategy [2].

## Conclusions

Lactation anaphylaxis represents a rare but important cause of recurrent life-threatening allergic reaction. Recognition of the breastfeeding trigger and cessation with the transition to formula feeding-or potentially prophylaxis with oral antihistamines-can be a lifesaving preventive measure. Lactation anaphylaxis should remain in the differential diagnosis and breastfeeding explored as a potential allergic trigger in any breastfeeding mother.
